# Identifying historical and future potential lake drainage events on the western Arctic coastal plain of Alaska

**DOI:** 10.1002/ppp.2038

**Published:** 2020-02-21

**Authors:** Benjamin M. Jones, Christopher D. Arp, Guido Grosse, Ingmar Nitze, Mark J. Lara, Matthew S. Whitman, Louise M. Farquharson, Mikhail Kanevskiy, Andrew D. Parsekian, Amy L. Breen, Nori Ohara, Rodrigo Correa Rangel, Kenneth M. Hinkel

**Affiliations:** ^1^ Water and Environmental Research Center University of Alaska Fairbanks Fairbanks Alaska; ^2^ Alfred Wegener Institute Helmholtz Centre for Polar and Marine Research Potsdam Germany; ^3^ University of Potsdam, Institute of Geosciences Potsdam Germany; ^4^ Department of Plant Biology University of Illinois Urbana Illinois; ^5^ Department of Geography University of Illinois Urbana Illinois; ^6^ Bureau of Land Management Arctic District Office Fairbanks Alaska; ^7^ Geophysical Institute University of Alaska Fairbanks Fairbanks Alaska; ^8^ Institute of Northern Engineering University of Alaska Fairbanks Fairbanks Alaska; ^9^ Department of Geology & Geophysics University of Wyoming Laramie Wyoming; ^10^ Department of Civil & Architectural Engineering University of Wyoming Laramie Wyoming; ^11^ International Arctic Research Center University of Alaska Fairbanks Fairbanks Alaska; ^12^ Michigan Technological University Houghton Michigan

**Keywords:** Arctic lakes, drained lake basins, lake drainage, permafrost regions, thermokarst lakes

## Abstract

Arctic lakes located in permafrost regions are susceptible to catastrophic drainage. In this study, we reconstructed historical lake drainage events on the western Arctic Coastal Plain of Alaska between 1955 and 2017 using USGS topographic maps, historical aerial photography (1955), and Landsat Imagery (ca. 1975, ca. 2000, and annually since 2000). We identified 98 lakes larger than 10 ha that partially (>25% of area) or completely drained during the 62‐year period. Decadal‐scale lake drainage rates progressively declined from 2.0 lakes/yr (1955–1975), to 1.6 lakes/yr (1975–2000), and to 1.2 lakes/yr (2000–2017) in the ~30,000‐km^2^ study area. Detailed Landsat trend analysis between 2000 and 2017 identified two years, 2004 and 2006, with a cluster (five or more) of lake drainages probably associated with bank overtopping or headward erosion. To identify future potential lake drainages, we combined the historical lake drainage observations with a geospatial dataset describing lake elevation, hydrologic connectivity, and adjacent lake margin topographic gradients developed with a 5‐m‐resolution digital surface model. We identified ~1900 lakes likely to be prone to drainage in the future. Of the 20 lakes that drained in the most recent study period, 85% were identified in this future lake drainage potential dataset. Our assessment of historical lake drainage magnitude, mechanisms and pathways, and identification of potential future lake drainages provides insights into how arctic lowland landscapes may change and evolve in the coming decades to centuries.

## 1 INTRODUCTION

Nearly one‐quarter of the lakes on Earth are located in the Arctic,^1^ although this proportion is probably an underestimate.^2^ The skewed distribution of lakes in the Arctic is largely dictated by past glaciation history, the presence of peat‐forming wetlands, and the presence of permafrost.^3^ Grosse et al.^4^ estimated that roughly 50% of the total lake area in permafrost regions occurs in soils with moderate (11% of permafrost region) to high (23% of permafrost region) ground ice content. The other half are found in areas with low (66% of permafrost region) ground ice content as mapped by the Circum‐Arctic map of permafrost and ground ice conditions.^5^ Lakes in permafrost regions can form as a result of permafrost degradation, ground ice melt, and terrain subsidence in areas with high to moderate ground ice content (i.e., thermokarst lakes that commonly form in areas with ice‐rich permafrost). Lakes may also develop in antecedent depressions, mainly in areas with low ground ice content, that interact with surrounding permafrost.^6^ The importance of permafrost‐region lakes to global climate change and northern high‐latitude soil and permafrost‐stored carbon cycling has been widely discussed,^7^, ^8^ yet determining how permafrost‐region lakes will respond to future projections of climate and land use change remains uncertain.^9^, ^10^ A better understanding of the distribution, timing, and processes associated with permafrost‐region lake drainage is important for carbon cycling,^7^ hydrology,^9^ hazards assessment, nutrient fluxes,^11^ water availability,^9^ tundra habitat and vegetation productivity,^11^, ^12^, ^13^ and permafrost dynamics.^14^, ^15^, ^16^, ^17^

Remote sensing time series image analysis has been widely used to document regional‐scale lake drainage patterns in permafrost regions.^18^, ^19^, ^20^, ^21^, ^22^, ^23^ The first such study by Mackay^24^ was based on a comparison of aerial photography, and showed that between 1950 and 1986 roughly 65 lakes had completely or partially drained, yielding a drainage rate of ~1.8 lakes/yr for the 5,000‐km^2^ study area located on the Tuktoyaktuk Peninsula, Northwest Territories, Canada. Hinkel et al.^18^ used Landsat satellite imagery acquired for one time period between ca. 1975 and ca. 2000 to analyze lakes larger than 10 ha for the western Arctic Coastal Plain (WACP) of northern Alaska (34,000 km^2^) and found that 50 lakes drained completely or partially (>25% reduction in surface area), or a drainage rate of ~2 lakes/yr. More recent observations have taken advantage of the increasing spatial and temporal resolution of remote sensing data to provide both more regionally robust lake drainage observations as well as finer temporally constrained lake drainage observations.^19^, ^20^, ^22^, ^25^, ^26^ Most recently, Nitze et al.^22^ presented a comprehensive lake change study for 10% of the permafrost region using dense Landsat imagery time series analysis and showed that lake drainage rates were highly variable spatially but that lake drainage was most concentrated along the transition between discontinuous and continuous permafrost zones.

The vulnerability of an individual lake to drain is dependent on the characteristics of the surrounding permafrost (ground ice content and distribution, ground temperature), lake characteristics (bathymetry, shore configuration, watershed and lake water balance), topography (the presence of a topographic drainage gradient and nearby erosional features such as streams, coasts and other lakes), climate (air temperature, precipitation and snow cover), and human activity.^24^ Common mechanisms that may lead to lake drainage in the continuous permafrost zone include ice‐wedge degradation (flow through ice‐wedge troughs), headward stream erosion, snow dam accumulation, bank overtopping, river channel migration, coastal erosion, underground piping or tunnel flow (drainage through open frost cracks or layers of permeable material), human disturbance, and expansion of a lake towards a drainage gradient.^4^, ^18^, ^19^, ^21^, ^24^, ^27^, ^28^, ^29^, ^50^

In spite of the basic understanding of how permafrost‐region lakes may drain,^24^ few studies have fully addressed the processes and pathways driving lake drainage, which limits our ability to project potential future lake drainage in the Arctic.^10^, ^18^, ^19^, ^29^While prior retrospective studies have yielded interesting results and provide useful information for evaluation of long‐ and short‐term landscape dynamics, they do not provide readily useful information for land and resource managers striving to plan for mitigating the impacts of climate and land use change in ice‐rich permafrost regions. Thus, an analysis that attempts to provide spatially explicit potential lake drainage information would provide context with which to make informed decisions for establishing infrastructure and water‐use activity plans. Furthermore, it would provide useful datasets for modeling carbon cycle feedbacks associated with permafrost‐region lakes^10^ and provide the opportunity to make important field observations prior to lake drainage that can further elucidate the processes driving catastrophic lake drainage in the Arctic.

In this study, we aim to answer the following primary questions: What are the spatial and temporal patterns of lake drainage via known drainage mechanisms? Further, can we identify lakes within a study region that appear most likely to drain in the near future based on historical lake drainage observations and landscape characteristics? To address these questions, we reconstruct lake drainage events for a 30,400‐km^2^ area on the WACP of Alaska between 1955 and 2017 using USGS topographic maps, historical aerial photography, Landsat MSS, Landsat TM, Landsat ETM+, and Landsat OLI datasets. Lakes that drained during the 62‐year study period were classified according to inferred drainage mechanism and drainage pathway. Drainage rates are then reported for three time periods (1955–1975, 1975–2000, and 2000–2017). Annual lake drainage analyses during the most recent period allowed us to identify common patterns associated with climate and ground ice content. In addition, we used a high‐resolution (5‐m) digital surface model (DSM) to identify the location of potential future lake drainage using lake‐specific drainage gradients, hydrologic connectivity, and historical observations. Our assessment of lake drainage rates and mechanisms, and identification of potential future lake drainages provides valuable information for studying how arctic lowland landscapes may respond and evolve as a result of climate and land‐use change over the coming decades to centuries.

## 2 STUDY AREA AND METHODS

**Figure 1:**
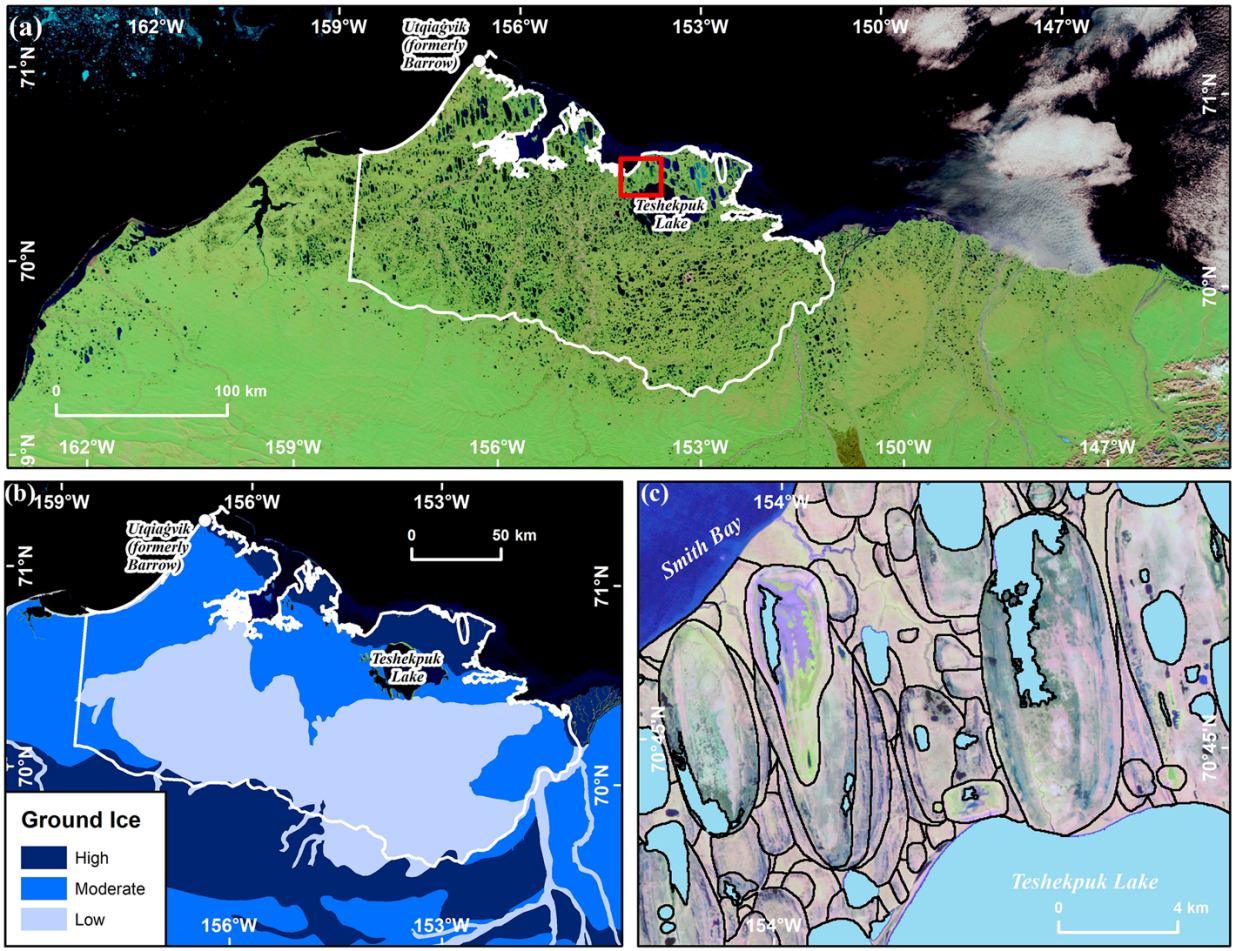
The historical and future potential lake drainage study region in northern Alaska. (a) Summer MODIS image of northern Alaska showing the numerous lakes that occur in the region. The 30,400‐km^2^ study region captures the majority of the WACP and is outlined by the white polygon. (b) Map showing the distinction between high, moderate, and low volumetric ground ice content terrain in surficial deposits of the study region.^39^ (c) Landsat TM image from 21 August 2010 showing numerous extant lakes (blue polygons) and the outline of numerous old and young drained thermokarst lake basins (hollow gray polygon) for a portion of the ice‐rich permafrost terrain north of Teshekpuk Lake. The location of panel (c) is depicted with a red box in (a) [Colour figure can be viewed at http://wileyonlinelibrary.com]

The 30,400‐km^2^ study area encompasses the majority of the lake‐rich WACP of Alaska (Figure 1).^30^ It represents the intersection of the lake drainage region analyzed by Hinkel et al.^18^ with the acquisition area of an airborne IfSAR (interferometric synthetic aperture radar)‐derived DSM acquired in 2002 and 2003 (Figure 1). Quaternary history has created a landscape with variable surficial geology, a range of ground ice content, and terrain where the distribution of thermokarst lakes and overlapping drained lake basins varies greatly.^31^ The southern region of our study area is underlain by ice‐poor eolian sand, while the northern regions are underlain by marine sand and silt with ground ice contents ranging from moderate to high (Figure 1). We identified all lakes larger than 10 ha that drained completely or partially (>25%) between 1955 and 2017 using historical (original) USGS topographic maps and aerial photography (1955) and Landsat Imagery (ca. 1975, ca. 2000, and annually since 2000). For each lake drainage event, we inferred the drainage mechanism and categorized the drainage pathway based on known lake drainage mechanisms^4^ using interpretation of high‐resolution remote sensing data and field observations. Finally, a future potential lake drainage product was developed using the IfSAR DSM data and refined with observations of historical lake drainages since 1955.

### 2.1 Historical lake drainages

**Figure 2:**
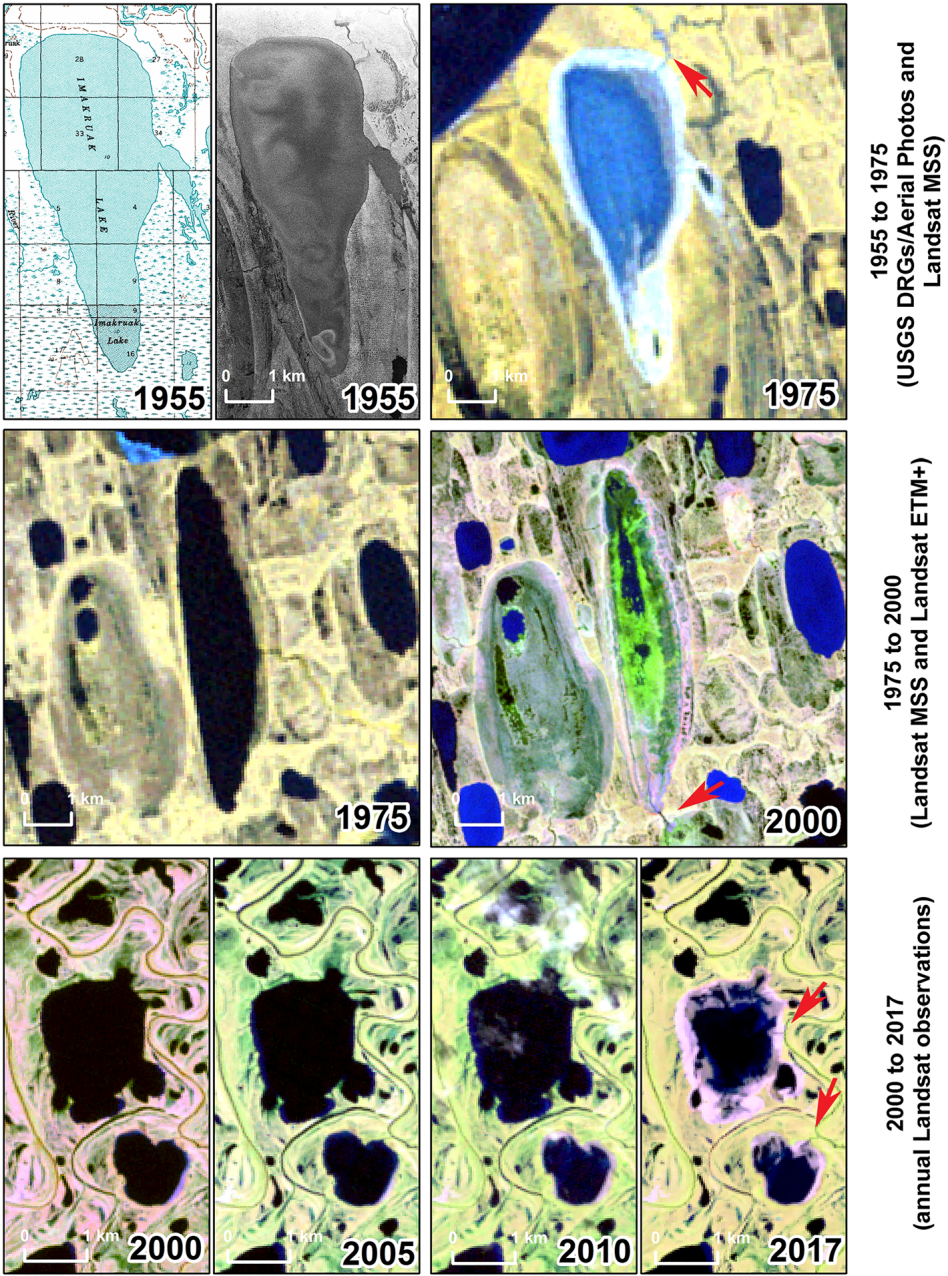
Remote sensing datasets and methods used to identify thermokarst lake drainages in three time periods between 1955 and 2017. Changes occurring in the first time period, 1955–1975, were mapped using USGS topographic maps and aerial photography from 1955 and Landsat MSS imagery from ca. 1975. Changes occurring in the second time period, 1975–2000, were mapped using Landsat MSS imagery and Landsat ETM+ imagery.^18^ Changes occurring in the last time period, 2000–2017, were mapped using time series analysis of Landsat TM, ETM+, and OLI imagery allowing for annual observations of lake drainage (note only four images are shown in the example). The location of the drainage pathway is shown with the red arrow in each example [Colour figure can be viewed at http://wileyonlinelibrary.com]

Methods used to identify historical lake drainages varied for each period and reflect improvements in the temporal resolution of image acquisition and processing capabilities over the past ~60 years (Figure 2). The first assessment period, 1955 to ca. 1975, involved comparison of original USGS topographic digital raster graphics created with 1955 era photography with the ca. 1975 lake area dataset derived from Landsat MSS data as presented by Hinkel et al.^18^ Lakes that potentially drained during this 20‐year period were manually identified. Each lake drainage event was further corroborated using the original black and white aerial photographs acquired in 1955 (1:50,000 scale) to eliminate identification of lakes that were incorrectly mapped in the 1955 topographic maps. The second assessment period, ca. 1975 to ca. 2000, is from Hinkel et al.^18^ Lake drainage during this 25‐year period involved remote sensing classification of lake surface water area in a Landsat MSS mosaic (ca. 1975) that was compared with a classification of lake surface area from a Landsat ETM+ mosaic (ca. 2000). Our study area is slightly smaller than the study area from Hinkel et al.^18^ due to the extent of the ca. 2002/03 IfSAR DSM, so we do not include 11 lake drainages that occurred on the WACP during this period. Lake drainages in the most recent assessment period, 2000–2017, were identified using all cloud‐free Landsat TM, ETM+, and OLI data observations in the study region following trend analysis methods presented by Nitze et al.^22^, ^25^ Annual observations of lake drainage derived from Landsat data enabled the identification of possible factors driving drainage over a 17‐year period on the WACP.

**Figure 3:**
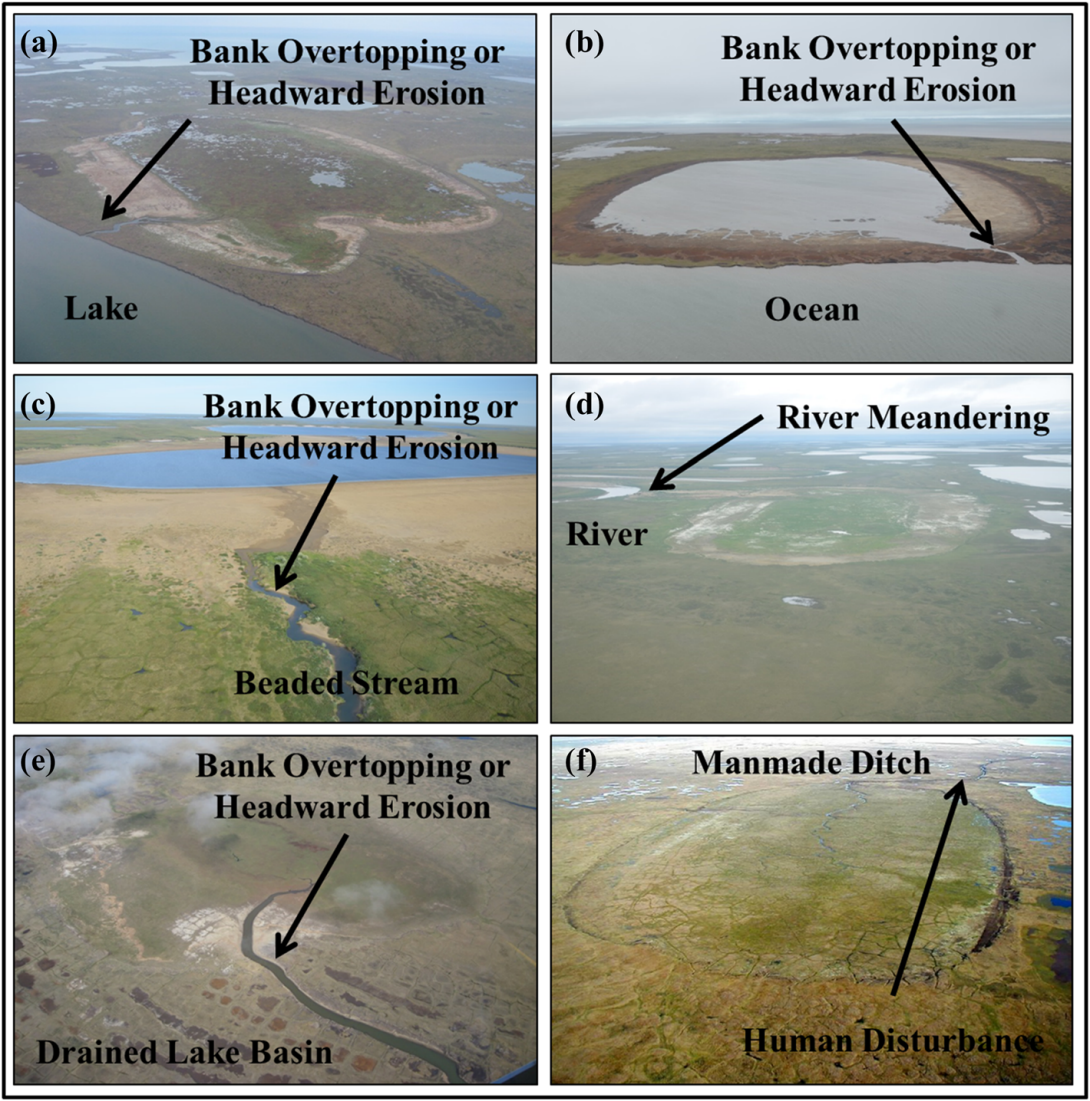
Oblique field photographs of lakes that drained on the WACP of northern Alaska during the study period with inferred drainage mechanisms and identification of various drainage pathways noted. These lakes drained (a) between 1955 and 1975, (b) between 2000 and 2017 and is now partially filled with ocean water, (c) between 1975 and 2000, (d) between 1975 and 2000, (e) between 1955 and 1975, and (f) between 1955 and 1975 [Colour figure can be viewed at http://wileyonlinelibrary.com]

**Figure 4:**
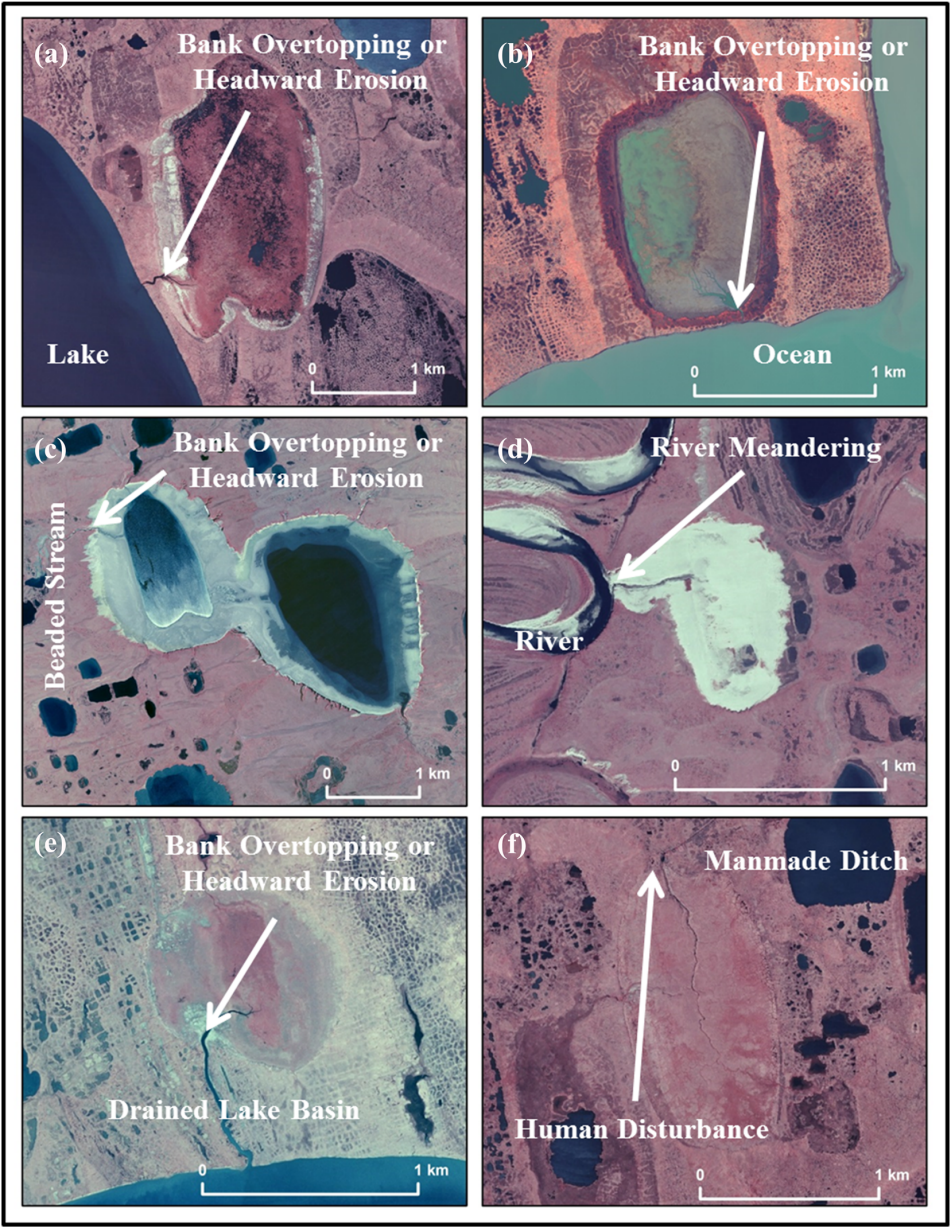
High‐resolution aerial photography (a, c–f: acquired between 2002 and 2007) and a Worldview‐2 satellite image (b: acquired in 2014) showing lake drainage pathways and inferred drainage mechanism. Lake examples mirror those provided in Figure 3 and are shown vertically to provide a sense of differences in scale and terrain features [Colour figure can be viewed at http://wileyonlinelibrary.com]

Every lake drainage event was further scrutinized using high‐resolution aerial photography or high‐resolution satellite imagery to infer the drainage mechanism and to identify the drainage pathway by using visual interpretation of high‐resolution imagery, combined with field site visits. This detailed assessment of each lake drainage event helped to reduce uncertainty associated with the detection of interannual variability in lake surface area relative to detecting changes caused by lateral lake drainage. The inferred drainage mechanisms follow methods by Hinkel et al.^18^ and Jones et al.^19^ and include bank overtopping or headward erosion, lake expansion, river meandering, coastal erosion, and human disturbance (Figures 3 and 4). Detailed analysis of each lake drainage event allowed for the categorization of every instance into an inferred mechanism category. This detailed assessment resulted in the removal of some possible lake drainage events from the dataset as it was apparent that some lakes did not have a drainage outlet and were probably changing their surface area unrelated to the geomorphic process of lake drainage. These lakes could have possibly changed surface area through processes related to drying, terrestrialization, or possibly lateral subsurface seepage. Lake drainage pathways represent the landscape feature that a particular lake drained into and include drained lake basins, beaded streams, lakes, rivers, the ocean, sloped tundra, and man‐made ditches (Figures 3 and 4).

In addition, the specific location of drainage was used to determine the tundra landform that probably facilitated lake drainage. Although the drainage location was readily identified using high‐resolution imagery, a small subset (<10 drained lakes) of historical drainage locations were difficult to identify optically due to decadal vegetation and geomorphic change. Therefore, local topographic and hydrologic gradients inferred from IfSAR DSM products were used to determine the likely point of drainage. We generated a 30‐m buffer (60 m in diameter) around each drainage outlet to extract tundra landforms from a Landsat‐derived landform map of the Arctic Coastal Plain of Alaska.^32^ However, thermoerosional gullies present at the drainage outlets probably inadequately reflect the surrounding tundra landforms,^32^ so we laterally shifted (~60 m) the drainage locations to accurately represent the actual tundra that probably facilitated drainage. We followed this protocol for both historical and potential future lake drainage.

### 2.2 Projecting potential future lake drainages

Assessment of lakes for their future potential to drain relied on the 2002/03 airborne IfSAR DSM data for the WACP. Lakes were extracted from this dataset using a slope derivative and manual correction.^33^ The vertical uncertainty for correctly detecting lake‐based drainage gradients with the IfSAR DSM was defined by comparing surface elevation differences of several overlapping DSM tile edges. This comparison showed standard deviations of elevation between overlapping IfSAR tiles ranging from 0.0 to 0.6 m. Thus, we chose a minimum height difference of 0.6 m to represent a detectable elevation gradient adjacent to a lake as being most likely to contribute to a rapid drainage event. This value is also in agreement with field‐verified estimates of the relative vertical accuracy (<0.5 m) of the DSM dataset around Utqiaġvik (formerly Barrow)^34^ and the stated vertical root mean square error (<1.0 m) of the DSM data.^35^

**Figure 5:**
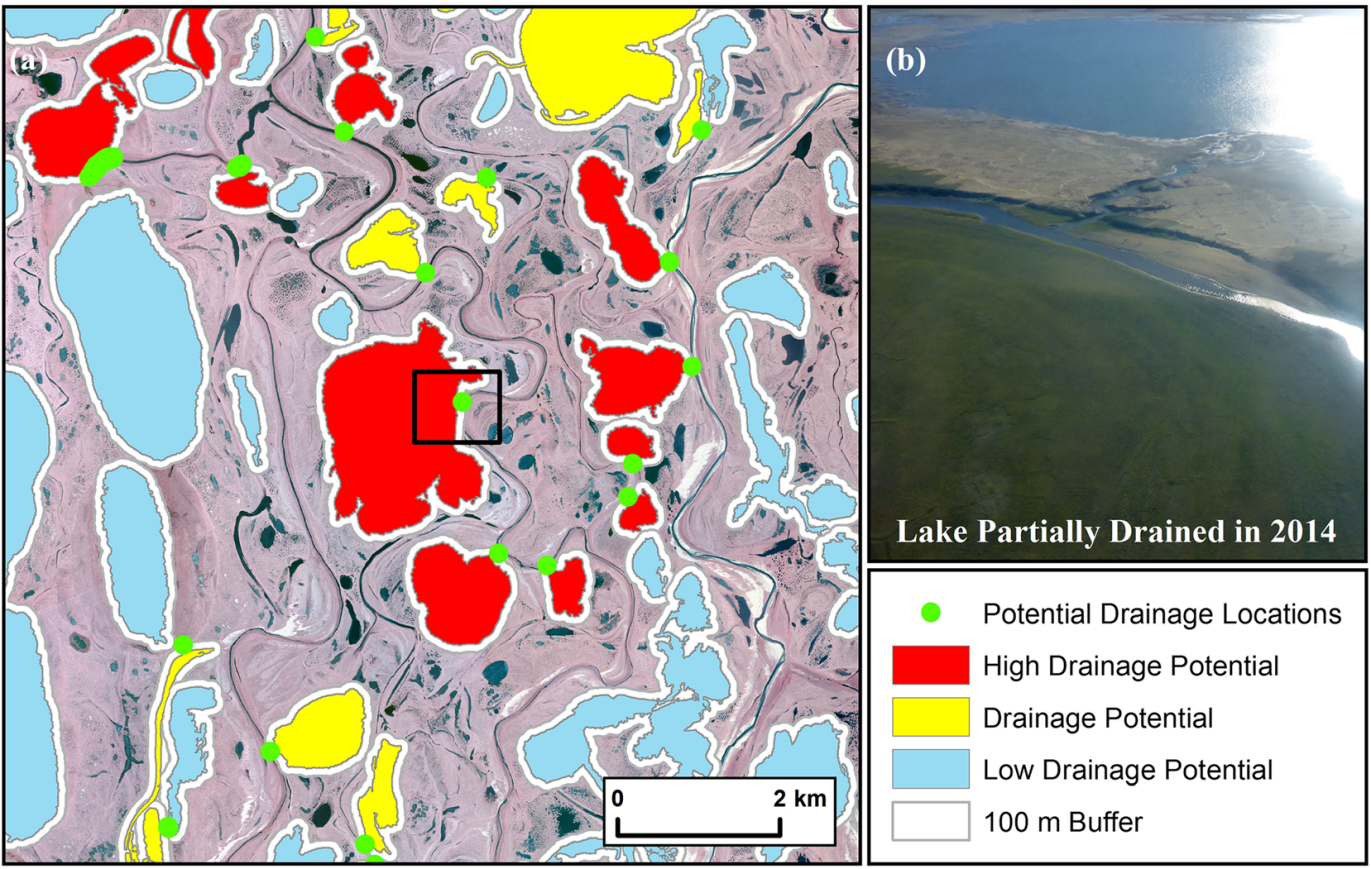
Locations of future potential lake drainage sites based on the potential lake drainage classification for a portion of the western Arctic coastal plain. (a) Lakes larger than 10 ha are color coded according to low drainage potential (blue), drainage potential (yellow), and high drainage potential (red) based on analysis of the IfSAR DSM data and manual categorization (high drainage potential). The green dots represent the location of the lowest point or series of points (5‐m pixel resolution) in the 100‐m buffer surrounding a specific lake if it met the drainage potential mapping criteria. (b) A ~350‐ha lake identified as having a high potential for drainage based on imagery acquired in 2002, partially drained (66% reduction in area) in 2014 (photo credit: CDA). The approximate view of the photograph taken in July 2017 is indicated with the black rectangle in (a) [Colour figure can be viewed at http://wileyonlinelibrary.com]

Development of the potential lake drainage dataset involved several processing steps. First, lakes were classified as potential future drainage candidates if the difference between the elevation of the lake surface and the lowest elevation within a 100‐m buffer of the lake shoreline exceeded our chosen threshold of 0.6 m. Next, we selected lakes with a minimum size of 10 ha to match the historical lake drainage dataset. We further filtered the dataset by selecting lakes estimated to have low hydrological connectivity based on relationships between lake contributing area as determined for specific surficial geology types and presented by Jones et al.^33^ This was added to the future projection workflow to isolate the lake population that probably responds to changes in surface area driven largely by geomorphic change as opposed to differences in surface hydrology. Lakes within a basin with low to no hydrologic connectivity that had an elevation change gradient between the lake surface and surrounding landscape are considered likely locations to assess for future drainage potential. Further, the greater the elevation difference, the greater the drainage potential. This dataset provided a first‐order estimate of lakes classified as being prone to future drainage. We further refined our assessment of potential drainage lakes by identifying the location of the point with the lowest elevation within the 100‐m buffer of the lake shoreline and manually interpreted lakes to have a high drainage potential based on the location of the probable drainage point to known lake drainage pathways using ca. 2002 orthophotography or more recent high‐resolution satellite imagery available for the WACP (Figure 5). Lakes classified as having a high drainage potential typically had the probable drainage location associated with one or more of the following: (a) an adjacent lake, (b) the cutbank of a river, (c) the ocean, (d) were located in an area with dense ice‐wedge networks, (e) appeared to coincide with a potentially headward eroding stream, or (f) were associated with thermokarst lake shoreline processes in the terrain of moderate to high ground ice content (Figure 1). We also added information on potential lake drainage pathways to the high potential drainage dataset by manually interpreting the landform associated with the probable drainage site to draw comparisons with the historical lake drainage dataset.

### 2.3 Short‐term climate datasets

Hydroclimatic data were acquired from the long‐term National Weather Service station at Utqiaġvik (station WBAN #700260, WMO #27502) from the Alaska Climate Research Center (http://akclimate.org/products) as mean daily values from 2000 to 2017, the period corresponding to annual resolution inventories of drainage events across the WACP. Summer air temperature (June–August) was used to evaluate variation in potential active layer deepening and ice wedge degradation to explain drainage frequencies. Total rainfall and end‐of‐winter snow depth (value on 1 May reported as snow‐water equivalent assuming a snow density of 250 kg/m^3^) were used to evaluate variation in runoff and its potential effects on mechanisms such as bank overtopping, headward erosion, and river meandering. Particularly with respect to rainfall and snow, it is understood that measurements on the Barrow Peninsula are only generally representative of the entire WACP. Denser networks of observations are necessary to more exactly understand variation in temperature and runoff and its role in lake drainage events, yet do not exist for these hydroclimate drivers of interest over this entire 18‐year period.

## 3 RESULTS

**Figure 6:**
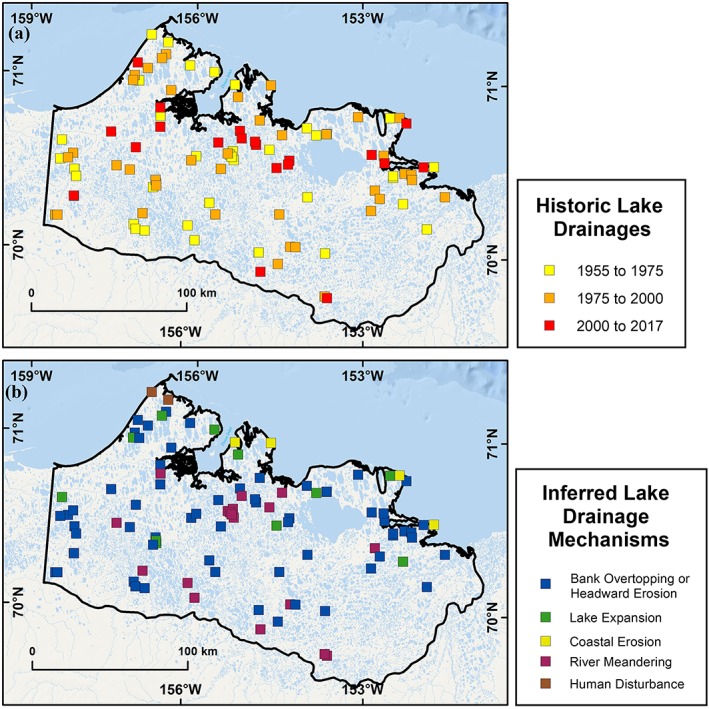
Historical lake drainages and inferred drainage mechanisms operating on the WACP between 1955 and 2017. (a) The drainage rate between 1955 and 1975 (yellow squares) was 2.0 lakes/yr, between 1975 and 2000 (orange squares) was 1.6 lakes/year, and between 2000 and 2017 (red squares) was 1.2 lakes/yr. (b) Inferred lake drainage mechanisms according to categories that include bank overtopping or headward erosion, lake expansion, coastal erosion, river meandering, and human disturbance [Colour figure can be viewed at http://wileyonlinelibrary.com]

Our multitemporal analysis of remotely sensed imagery identified 98 lake drainage events (lake of >10 ha and more than 25% reduction in lake area) of the more than 7,600 lakes on the WACP between 1955 and 2017, which equates to a historical (62‐year) drainage rate of 1.6 lakes per year in the 30,000‐km^2^ study area (Figure 6). Spatially, lake drainage patterns occurred in all regions of ground ice content (high, moderate, and low) (Figure 1b) but the number of lake drainages in a given period fluctuated over time. We explore the spatial and temporal patterns of historical and future potential lake drainage in more detail below.

### 3.1 Lake drainage events between 1955 and ca. 1975

Between 1955 and ca. 1975, we identified 39 lakes that drained on the WACP, or an average drainage rate of 2.0 lakes per year (Table 1). During this period, 31 lakes drained completely (>95% area reduction) and eight lakes drained partially (>25% area reduction). The majority of the lake drainages occurred in moderate (*n* = 17) and low (*n* = 15) ground ice content terrain relative to the high (*n* = 7) ground ice content terrain (Table 2). Of the 39 lake drainages, the majority (*n* = 21) were inferred to have drained as a result of bank overtopping or headward erosion, eight as a result of river meandering, six as a result of lake expansion, and two each as a result of coastal erosion and human activity. The primary drainage pathways consisted of adjacent drained lake basins (*n* = 12), rivers (*n* = 9), and lakes (*n* = 7). Beaded streams (*n* = 5), man‐made ditches (*n* = 3), the ocean (*n* = 2), and sloped tundra (*n* = 1) were less frequent pathways for lake drainage.

**Table 1:** Lake drainages, drainage rates (lakes/yr), drainage pathway, and inferred drainage mechanism for three time periods between 1955 and 2017. Numbers in parentheses are percentages

			Drainage pathway							Drainage mechanism				
Time period	Number of drained lakes	Lake drainage rate	Drained lake basin	Beaded stream	Lake	River	Ocean	Sloped tundra	Man‐made ditch	Bank overtopping or headward erosion	Lake expansion	River meandering	Coastal erosion	Human disturbance
1955–1975	39	1.95	12 (31)	5 (13)	7 (18)	9 (23)	2 (5)	1 (3)	3 (8)	21 (54)	6 (15)	8 (21)	2 (5)	2 (5)
1975–2000	39	1.56	15 (38)	5 (13)	6 (15)	5 (13)	2 (5)	6 (15)	0 (0)	26 (67)	4 (10)	7 (18)	2 (5)	0 (0)
2000–2017	20	1.18	5 (24)	1 (5)	1 (5)	10 (48)	2 (10)	1 (5)	0 (0)	16 (80)	1 (5)	3 (14)	0 (0)	0 (0)

**Table 2:** Lake drainage mechanism in each of the three study time periods relative to high, moderate, and low volumetric ground ice content in permafrost terrain derived from Jorgenson et al^39^

	Drainage mechanism											
Time period	Bank overtopping or headward erosion			Lake expansion			River meandering			Coastal erosion		
	High	Mod	Low	High	Mod	Low	High	Mod	Low	High	Mod	Low
1955–1975	3	9	11	3	1	2	0	6	2	1	1	0
1975–2000	3	12	12	1	2	1	0	2	5	1	0	0
2000–2017	2	13	1	0	1	0	0	1	2	0	0	0

### 3.2 Lake drainage events between ca. 1975 and ca. 2000

Our updated assessment builds upon the previous results reported by Hinkel et al.^18^ by restricting the study area presented in this paper and by reinterpreting some of the lake drainage mechanisms based on the use of high‐resolution orthoimagery as well as adding the lake drainage pathway criterion. Between ca. 1975 and ca. 2000, we identified 39 lakes that drained on the WACP, or a drainage rate of 1.6 lakes/yr. During this period, 19 lakes drained completely and 20 lakes drained partially. The majority of the lake drainages occurred in moderate (*n* = 16) and low (*n* = 18) ground ice content terrain relative to the high (*n* = 5) ground ice content terrain (Table 2). Of the 39 lake drainages, the majority (*n* = 26) were inferred to have drained as a result of bank overtopping or headward erosion, seven as a result of river meandering, four as a result of lake expansion, two as a result of coastal erosion, and none as a result of human activity. The primary drainage pathway consisted of adjacent drained lake basins (*n* = 15). Rivers (*n* = 5), lakes (*n* = 6), beaded streams (*n* = 5), and sloped tundra (*n* = 6) all accounted for roughly the same number of lake drainage pathways whereas the ocean (*n* = 2) was a less frequent pathway for drainage.

### 3.3 Lake drainage events between ca. 2000 and 2017

Between ca. 2000 and 2017, we identified 20 lakes that drained on the WACP, or an average drainage rate of 1.2 lakes/yr. During this period, three lakes drained completely and 17 lakes drained partially. The majority of the lake drainages occurred in moderate (*n* = 15) ground ice content terrain relative to the high (*n* = 2) and low (*n* = 3) ground ice content terrain. Of the 20 lake drainages, the majority (*n* = 16) were inferred to have drained as a result of bank overtopping or headward erosion, three as a result of river meandering, one as a result of lake expansion, and none as a result of coastal erosion or human activity. The primary drainage pathways consisted of rivers (*n* = 10). Drained lake basins (*n* = 5), lakes (*n* = 1), the ocean (*n* = 2), beaded streams (*n* = 1), and sloped tundra (*n* = 1) were less frequent drainage pathways.

Detailed time series analysis of the Landsat archive between 2000 and early July 2017 showed annual variability in lake drainage patterns (Table 3). No lake drainage events were detected in the years 2000, 2002, 2003, 2005, 2007, 2009–2011, and 2015–2017. Thus, the 20 lake drainage events detected were distributed in eight of the 17 years. For the years in which lake drainage events were detected, 11 events occurred in 2004 (*n* = 5) and 2006 (*n* = 6). Two lake drainages occurred in 2001, 2012, 2013, and 2014, while one occurred in 2008. Bank overtopping and/or headward erosion was inferred to be the sole drainage mechanism in 2004 and accounted for 83% of the drainages in 2006.

**Table 3:** Detailed analysis of lake drainages, drainage pathways, and drainage mechanisms based on Landsat trend analysis from 2000 to 2017

		Drainage mechanism				Drainage pathway					
Year	Number of drained lakes	Bank overtopping or headward erosion	Lake expansion	River meandering	Coastal erosion	Drained lake basin	Beaded stream	Lake	River	Ocean	Sloped tundra
2000	–	–	–	–	–	–	–	–	–	–	–
2001	2	2	–	–	–	–	–	–	2	–	–
2002	–	–	–	–	–	–	–	–	–	–	–
2003	–	–	–	–	–	–	–	–	–	–	–
2004	5	5	–	–	–	2	1	–	1	–	1
2005	–	–	–	–	–	–	–	–	–	–	–
2006	6	5		1		1		1	3	1	
2007	–	–	–	–	–	–	–	–	–	–	–
2008	1	–	–	1	–	–	–	–	1	–	–
2009	–	–	–	–	–	–	–	–	–	–	–
2010	–	–	–	–	–	–	–	–	–	–	–
2011	–	–	–	–	–	–	–	–	–	–	–
2012	2	2	–		–	1	–	–	1	–	–
2013	2	1	–	1	–	1	–	–	1	–	–
2014	2	2	–	–	–	–	–	–	1	1	–
2015	–	–	–	–	–	–	–	–	–	–	–
2016	–	–	–	–	–	–	–	–	–	–	–
2017	–	–	–	–	–	–	–	–	–	–	–

### 3.4 Lake drainage events relative to permafrost and landscape characteristics

Lake drainages between 1955 and 2017 were distributed across three primary terrain units, which vary with respect to land area, lake density, and ground ice content (high, medium, and low). The land area of the high ground ice content region was 2,833 km^2^, the moderate ground ice region was 10,158 km^2^, and the low ground ice content region was 16,434 km^2^. The density of lakes in these three regions varied from 0.15, 0.24, and 0.30 lakes/km^2^, respectively. Thus, the relative impact of lake drainage on the lake population in a given region was highest for the high ground ice content terrain (3.5%), second highest for the moderate ground ice content region (1.9%), and lowest for the low ground ice content region (0.7%) over the 62‐yr period. In terms of an area‐normalized lake drainage rate (land area/10,000 km^2^) among the three terrain units, the high ground ice content region had 0.85 lakes drain/yr/10,000 km^2^, the moderate ground ice content region had 0.76 lakes drain/yr/10,000 km^2^, and the low ground ice content region had 0.35 lakes drain/yr/10,000 km^2^.

An analysis to determine the tundra landforms that probably facilitated lake drainage identified well‐defined ice‐wedge polygonal landforms to be important avenues for gully formation and historical drainage. Overall, more than 50% of the gullies that facilitated lake drainage were associated with high‐centered ice‐wedge polygonal terrain, 30% with low‐centered polygonal terrain, and the remaining 20% were distributed among flat‐centered polygonal terrain, drained slopes, non‐patterned drained lake basins, and ponds. Drainage through high‐centered polygonal terrain dominated (58%) the inferred bank overtopping and headward erosion mechanisms. Lakes that drained via coastal erosion and river meandering were more commonly associated with low‐centered ice wedge polygonal terrain (~50%) and lakes that drained in response to adjacent lake expansion occurred in a mixture of high‐centered (48%) and low‐centered (24%) polygonal landform types.

### 3.5 Potential future lake drainage sites

**Figure 7:**
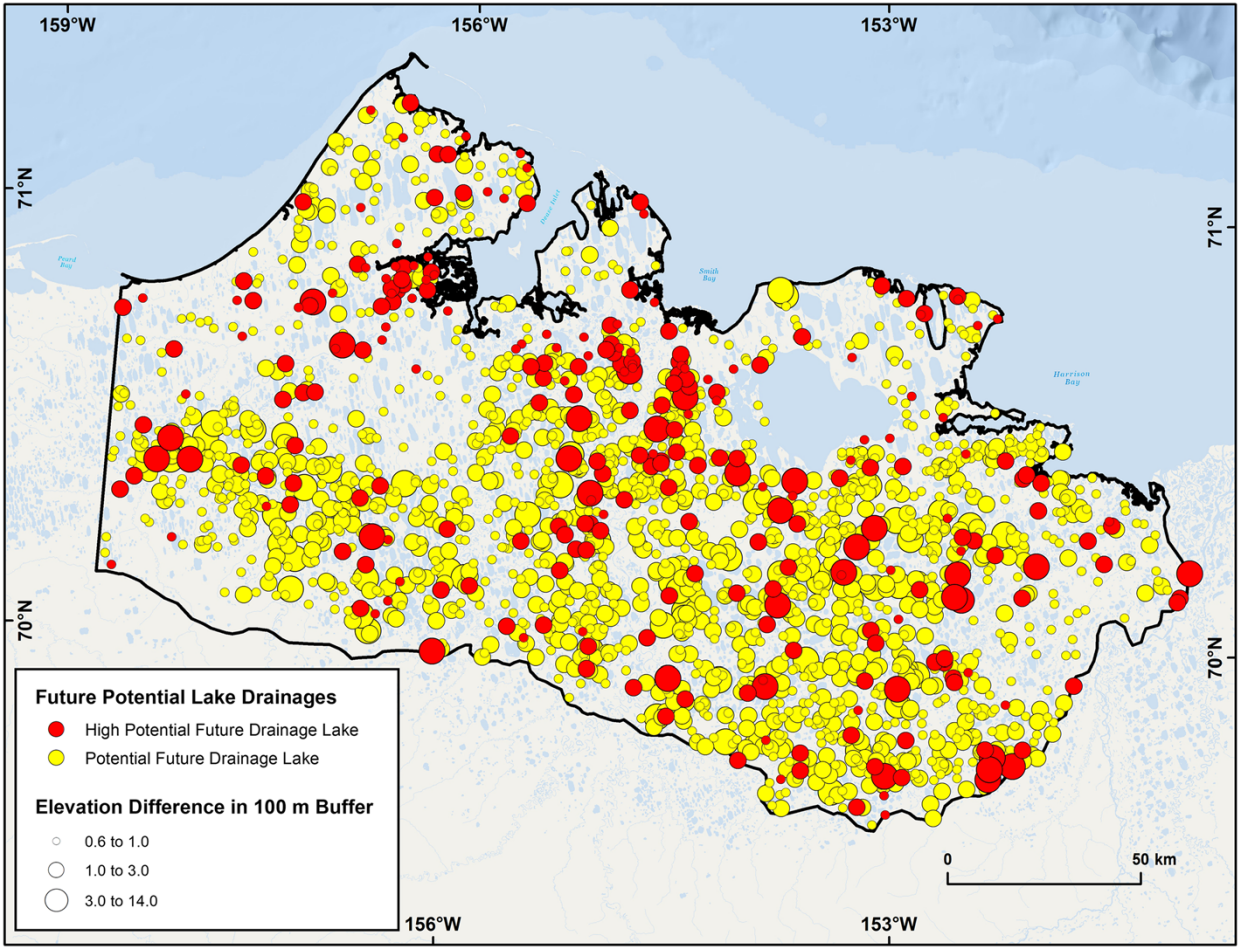
Lakes (>10 ha) identified as being susceptible to future drainage based on low hydrologic connectivity and an elevation change greater than 0.6 m within 100 m of the shoreline. This analysis detected 25% of the 7,630 lakes (>10 ha) on the WACP (yellow circles). Lakes manually identified as having a high potential for near‐future drainage are also shown (red circles). Detectable elevation changes range from 0.6 to 14.0 m and are shown as a three category graduated circle (0.6–1.0 m, 1.0–3.0 m, 3.0–14.0 m) [Colour figure can be viewed at http://wileyonlinelibrary.com]

**Figure 8:**
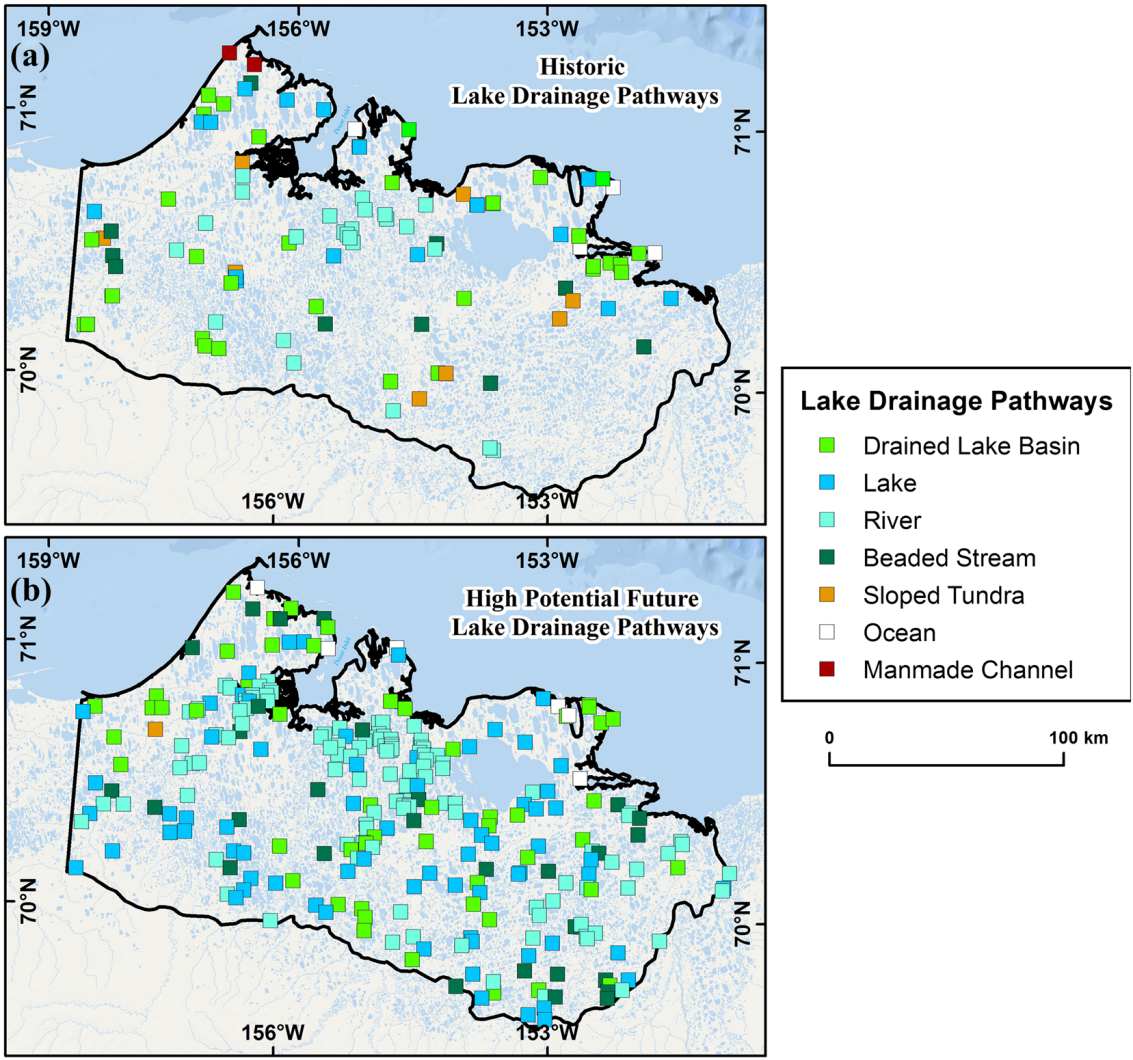
Historical and potential future lake drainage pathways. (a) Historical lake drainage pathways between 1955 and 2017. This represents the landscape feature that a particular lake drained into. (b) Potential future lake drainage pathways based on the IfSAR DSM and manual categorization of lakes of high potential for drainage and the landscape feature where the drainage potential location was located. This information can be used to identify and instrument lakes likely to drain and highlight their probable inferred mechanism for studying future landscape evolution on the WACP [Colour figure can be viewed at http://wileyonlinelibrary.com]

We extracted 7,630 lakes with a surface area greater than 10 ha from the 2002/03 IfSAR DSM dataset. Of these, 2,748 lakes had an elevation difference exceeding 0.6 m below water level, within 100 m of the shoreline. A buffer of 100 m was used to represent the average drainage gully length associated with observations of historical drainage in the region. Based on the criteria for determining limited hydrological connectivity in varying surficial geology types, 1,934 (25%) lakes were identified as being susceptible to future drainage (Figure 7). We manually identified the probable drainage pathway for each of these lakes and found that adjacent drained lake basins accounted for 48%, lakes for 18%, rivers for 15%, beaded streams for 11%, sloped tundra for 6% and the ocean for less than 0.5% of the probable pathways. Of the 1,934 lakes identified as being potentially prone to drainage, we manually identified 275 (14%) as having a high drainage potential based on the elevation difference in the area around the lake as well as the landscape setting of the potential drainage location (Figures 5 and 7). Analysis of the 275 lakes identified as having a high drainage potential showed that rivers accounted for 43%, lakes 25%, drained lake basins 19%, beaded streams 11%, the sea 2%, and sloped tundra sites less than 0.5% (Figure 8).

The 1,934 potential future lake drainage candidates are distributed throughout the study region. The majority of the lakes (65%) are located in the low ground ice content region, while 30% are located in the moderate ground ice region and 5% in the high ground ice content region. When standardizing these numbers relative to the lake number in each region, this equates to 25%, 23%, and 22%, respectively. Thus, the relative portion of potential future lake drainage events in each ground ice type region is fairly evenly distributed. The mean difference in elevation between the lake surface and the lowest point in the 100‐m buffer around a particular lake is lowest in the high ground ice content region (0.98 m) and highest in the low ground ice content region (1.40 m), with the moderate ground ice content region being in between (1.08 m). The maximum elevation change adjacent to a lake also follows this pattern, ranging from high ground ice content (3.6 m), moderate ground ice content (4.5 m), to low ground ice content (13.8 m).

To test our lake drainage potential dataset, we compared drainage‐potential lakes in the 2002/03 IfSAR DSM with mapped lake drainage events from our 2000–2017 remote sensing observation period. Of the 20 lakes that drained between 2000 and 2017, 17 (85%) were identified as a probable drainage candidate in the IfSAR‐based assessment. The three lakes that we failed to capture in our database had an elevation difference less than the 0.6‐m threshold that we used to identify drainage potential lakes, which was constrained by the quality of the DSM. Of the 17 lakes that drained and were mapped as having a potential to drain, 12 (71%) were manually interpreted as having a high potential to drain based on the manual assessment of lake drainage candidates. Three of these five lakes were actually initially drained in an earlier period (e.g. 1975–2000) but continued to drain in the latter period (2000–2017) and were thus already considered to be partially drained. Two lakes were simply not identified as having a high potential to drain and highlight some limitations of our lake drainage projection approach.

## 4 DISCUSSION

Permafrost region lake drainage has long been viewed as an important landscape change mechanism across the Arctic.^28^ Evidence of thermokarst lake drainage is seen in the abundance of drained lake basins covering >60% of arctic lowlands in northern Alaska, Russia, and Canada.^4^ Our analysis updates lake drainage rates and timing for a well‐studied arctic region and provides added information on lake drainage mechanisms and pathways that can be used to inform a dataset created to anticipate the location of future lake drainage candidates.

### 4.1 Decadal‐scale lake drainage rates

We identified a decrease in the decadal‐scale lake drainage rate between 1955 and 2017 on the WACP, declining by 20% between the first and second period and by an additional 24% between the second and third period. Thus, it appears that the conditions conducive to lake drainage and/or the long‐term transient effects that eventually result in lake drainage were more favorable between 1955 and ca. 1975 than they have been since that time. If lakes in the region are trending towards a state whereby future lake drainages are occurring less frequently, it could result in more enhanced talik development due to a longer “lake lifespan” and therefore potentially increase ancient C release. 10, 36However, the apparent decrease in lake drainage in the WACP study region may simply reflect the long‐term transient nature of this active landscape‐scale process.

We compared the reduction in decadal‐scale drainage rates that we identify on the WACP of northern Alaska with similar studies conducted for other regions in Alaska and Canada by normalizing the observations according to the number of lakes drained per year per 10,000 km^2^ (Table 4). Our observations show that lake drainage declined from 0.65 (1955–1975), to 0.52 (1975–2000) and to 0.39 (2000–2017) lakes/yr/10,000 km^2^ for the WACP of northern Alaska. This normalized rate decline in lake drainage is similar to that found on the Tuktoyaktuk Coastlands in Canada, which showed a reduction from 0.77 lakes/yr/10,000 km^2^ from 1950 to 1973 to 0.33 lakes/yr/10,000 km^2^ from 1985 to 2006.^21^ These observations of declining lake drainage in relatively cold permafrost regions are in contrast to an increase in lake drainage rates in the warm permafrost region of the Old Crow Flats where lake drainage increased from 0.36 to 2.02 lakes/yr/10,000 km^2^ between 1951–1972 and 1972–2010, respectively.^20^ Area‐normalized lake drainage rates for the warmer permafrost region of the northern Seward Peninsula in Alaska showed sustained and high lake drainage rates of 7.52 lakes/yr/10,000 km^2^ between 1950 and 2007.^19^ These contrasting patterns of area‐normalized lake drainage rate decreases and increases require further study across larger regions and over longer time‐scales.

**Table 4:** Comparison of area‐normalized lake drainage rates for several study regions between 1950 and 2017

Study	Study region	Time period	Lake drainages (*n*)	Normalized study area (area/10,000 km^2^)	Area‐normalized lake drainage rate^a^
24	Tuktoyaktuk coastlands, Canada	1950–1986	65	0.50	2.31
18	Western Arctic Coastal Plain of northern Alaska	1975–2000	50	3.45	0.57
21	Tuktoyaktuk coastlands, Canada	1950–1973	26	1.00	0.77
21	Tuktoyaktuk coastlands, Canada	1973–1985	10	1.00	0.83
21	Tuktoyaktuk coastlands, Canada	1985–2006	7	1.00	0.33
19	Northern Seward peninsula, Alaska	1950–2007	30	0.07	7.52
29	Western Arctic Coastal Plain of northern Alaska	1955–2014	9	0.18	0.87
20	Old Crow Flats, Canada	1951–1972	4	0.56	0.36
20	Old crow flats, Canada	1972–2010	34	0.56	2.02
This study	Western Arctic Coastal Plain of northern Alaska	1955 to ca. 1975	39	3.00	0.65
This study	Western Arctic Coastal Plain of northern Alaska	ca. 1975 to ca. 2000	39	3.00	0.52
This study	Western Arctic Coastal Plain of northern Alaska	ca. 2000 to 2017	20	3.00	0.39

a Area‐normalized lake drainage rate refers to the number of lake drainages per year divided by the normalized study area

### 4.2 Annual‐scale lake drainage events and weather patterns

**Figure 9:**
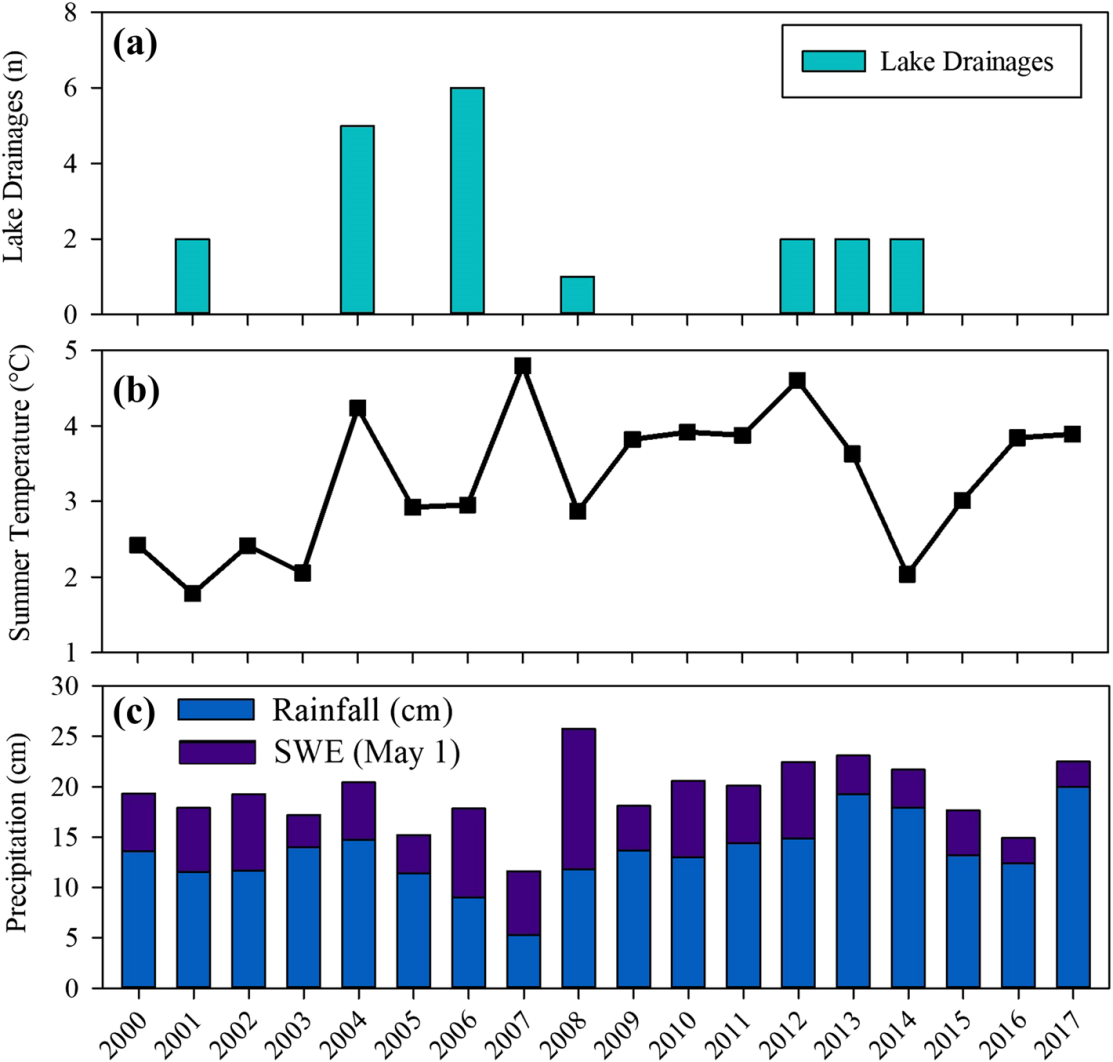
(a) The number of lake drainage events, (b) the mean summer (June, July, and August) air temperature, and (c) annual summer and winter precipitation totals between 2000 and 2017 [Colour figure can be viewed at http://wileyonlinelibrary.com]

Our annual‐scale analysis of lake drainage events between 2000 and 2017 showed that 55% of the lakes that drained during this time drained in just two of the 17 years. This is important for two reasons: first, it provides information on the potential temporal variability in lake drainage associated with assessment of this process over decadal time scales and, second, it provides the observational data necessary to identify potential external factors that contribute to temporally clustered lake drainage events. Jones and Arp^29^ documented the conditions that promoted a thermokarst lake to drain near the Beaufort Sea coast in 2014, in the region with high volumetric ground ice content. In this case, the combination of a rapidly eroding ocean coastline over the course of the last several decades and abundant winter snowfall and heavy early summer precipitation in 2014 resulted in elevated lake water levels that probably promoted bank overtopping and thermo‐erosion along an ice‐wedge network. Our dataset showed that a second lake also drained during 2014, apparently through the same bank overtopping/thermo‐erosion mechanism. During 2004 and 2006, five and six lakes drained (Figure 9), respectively, probably due to bank overtopping and/or headward erosion, accounting for 85% of the drainage events between 2000 and 2017 (Table 3).

We assessed the summer air temperature and summer and winter precipitation relative to the annual time series of lake drainage events in the study region (Figure 9). At first glance, there does not appear to be much of a relationship between external forcing conditions and the annual lake drainage data. The summer of 2004 was notably warm and relatively wet but the summer of 2006 was about average in terms of temperature and precipitation. Other subsequent years seemingly had similar conditions to 2004 and 2006, yet no or only one or two lakes drained. One explanation for this could be that the drought conditions present in the summer of 2007 caused lake level declines in the region.^9^, ^37^, ^38^ Thus, in spite of a series of consecutive wet and relatively warm years, lake levels are probably still responding to the drawdown in 2007. Based on the clustering of lake drainage events that occurred in 2004 and 2006, it appears that a period of enhanced lake drainage might occur in the next few years given progressively warm and wet conditions shown in the weather station data. These findings highlight, for example, that “externally forced (short term)” and “intrinsic cycling (long term)” processes and factors are acting together across various spatial and temporal scales. Parsing these confounding lake drainage factors will require future detailed field observations of lake drainages and potential lake drainage candidates.

### 4.3 Complete vs. partial lake drainage processes and pathways

There has been an apparent shift in the nature of lake drainage from complete to partial over time. Between 1955 and ca. 1975, 80% of the lake drainages involved complete loss of water in the basin. Between ca. 1975 and 2000, 49% of the lakes drained completely and 51% of the lakes drained partially. Between 2000 and 2017, only 14% of the lake drainages resulted in complete drainage. This apparent shift from complete to partial drainage could be due to a number of factors such as increased active layer seepage, increased precipitation, the type of lakes draining, and/or differences in the lake drainage pathway (i.e., lake draining into a river, lake, or ocean that then floods the basin post‐drainage). A detailed assessment of this potentially interesting shift in lake drainage dynamics requires further field study as it may have implications for carbon cycling, hydrology, hazards, and permafrost dynamics.

### 4.4 Lake drainage patterns relative to surficial geology

Our findings show that lake drainage may occur in regions with high, moderate, and low ground ice content and show that ground ice content is not necessarily a key factor in controlling the likelihood of drainage (Table 2). However, based on the distribution of lakes greater than 10 ha according to surficial geology, the greatest relative impact of lake drainage occurred in high ground ice content terrain with a loss of 4.5% of the lakes larger than 10 ha in that region. This is in comparison to a loss of 2.0% and < 1.0% for the moderate and low ground ice content regions, respectively. These patterns also hold for the greatest impact relative to the respective land area in each of the three primary ground ice content regions, with the density of lake drainage events (lakes/yr/10,000 km^2^) being highest in the high ground ice content region and lowest in the low ground ice content region. Overall, patterns of lake drainage in regions with variable ground ice contents and lake origins highlight the important role of lake–permafrost interactions and lake drainage in spite of whether lake formation and/or drainage is initiated through thermokarst and/or thermal erosion or other means in permafrost regions.

### 4.5 Lake drainage mechanisms and future drainage implications

Hopkins^28^ described that the probable drainage of lakes on the Seward Peninsula resulted from degradation of ice wedges that extended from the lake shore into a lower lying adjacent area. Given an increase in Pan‐Arctic ice wedge degradation,^39^, ^40^ this process might become more important in the future. Catastrophic lake drainage via ice‐wedge degradation has also been described for many lakes in Canada on the Tuktoyaktuk Peninsula^24^ and on the Arctic Coastal Plain of northern Alaska^29^ but the process most likely to have caused the drainage in our study was bank overtopping and thermo‐erosional gully formation, which also tends to occur due to flow along ice wedge troughs. The headward erosion of streams on the Barrow Peninsula was observed by Lewellen^41^ to rapidly respond to extreme precipitation events, as two streams with no knickpoint migration between 1956 and 1962 eroded 55 m of tundra during the rainy summer of 1963. Rapid development of a new drainage system was also observed in the Canadian Arctic on Bylot Island, where a 750‐m‐long gully system was developed along the ice‐wedge network in four years with maximum headward erosion rates of up to 5 m/day.^42^ Given increases in coastal erosion in the region, lakes located near the Beaufort and Chukchi Sea might become more vulnerable to drainage or lagoon formation in the future.^43^ Future field efforts should focus on observing lakes prior to, during, and immediately following rapid drainage to provide critical information to inform future assessments of landscape changes in the Arctic.

Our interpretations of lake drainage mechanisms were further refined by taking into account the estimated volumetric ground ice content of the terrain^39^ in which lakes drained. Our assessment of the location of historical lake drainages showed that lakes preferentially drained into areas with high‐centered polygonal tundra and low‐centered polygonal tundra as derived from Lara et al.^12^, ^32^ As stated previously, bank overtopping and/or headward erosion were identified as the dominant (64%) drainage mechanism for the entire study period as well as for each of the three time periods. Despite observational evidence suggesting lake expansion may be the dominant mechanism for drainage, 79% of all drained lakes in our study region occurred in terrain not conducive to rapid themokarst lake expansion (low to moderate ground ice content), indicating this drainage mechanism may be regionally specific and not prevalent across the WACP. In each of these previous studies,^18^, ^19^ bank overtopping and/or headward erosion were identified as the second leading mechanism of lake drainage. There was a consistent pattern of lake drainage by bank overtopping and/or headward erosion in the high ground ice content terrain, a potential increase of this mechanism in the moderate ice content terrain, and a notable decline (from 11 and 12 to 1 during the three study periods, respectively) in the low ground ice content terrain (Table 2). The large decrease in the number of lake drainages occurring in the low ground ice content terrain during the third period is the primary reason that the decadal‐scale, landscape assessment of lake drainage rates declined by more than 20% between 2000 and 2017.

**Figure 10:**
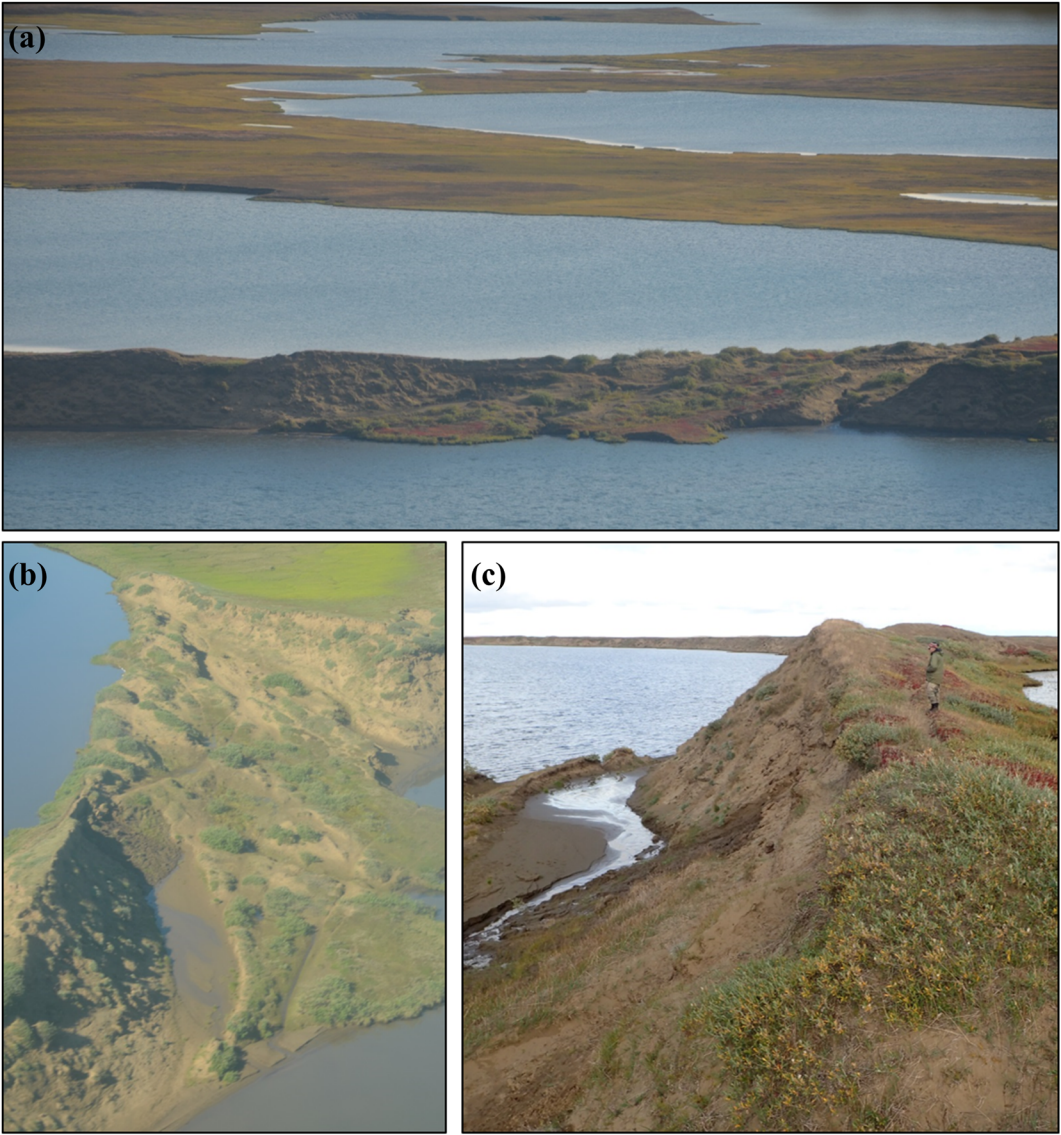
Example of a lake (informally known as P Lake) with a high drainage potential. P Lake (70.213893°, −153.313524°) is 9.2 m above the adjacent lake and subsurface seepage occurring in a sand dune blowout is probably contributing to maintaining the lake level and potentially preventing it from overtopping its bank. However, this process might eventually lead to its drainage through sapping [Colour figure can be viewed at http://wileyonlinelibrary.com]

Lakes in the eolian sand study area are not considered to be lakes formed by thermokarst due to insufficient ground ice content in the permafrost to promote thaw subsidence following degradation.^6^ However, permafrost is continuous in this region and the lakes do interact with the surrounding permafrost terrain through drainage processes. We propose two primary hypotheses for the disproportionate decline in lake drainage in the low ground ice content region: (a) the climate gradient that exists between the coast and inland on the ACP is not responding uniformly to climate changes (temperature, precipitation, and/or snow) that may have occurred since the early 2000s as a result of sea ice decline^44^, ^45^; and (b) warming in the region is impacting the low ground ice content terrain through increased active layer thickness and promoting an increase in subsurface and semi‐lateral seepage that is limiting the drainage of lakes in spite of increases in precipitation in the summer and the winter.^9^ We provide an example of the latter from a lake located in the surficial geology unit composed of eolian sand with low ground ice content (Figure 10). In our example, P Lake (informal name) sits 9 m above the adjacent lake and subsurface seepage occurring in a sand dune blowout is probably preventing the lake from overtopping the narrow isthmus.

## 5 INFORMING FUTURE LANDSCAPE CHANGE STUDIES

Few studies have attempted to identify future lake drainage patterns at the landscape or local scale.^10^, ^36^, ^46^ These studies have primarily used modeling to approach this question. While lake drainage on the WACP appears to be decreasing at the decadal scale, our analysis indicates the importance of particular factors that might contribute to periods of enhanced lake drainage. Lake drainage is increasing and already occurring more rapidly in areas with warmer permafrost than the WACP.^20^, ^22^ Thus, it is conceivable that lake drainage may increase in the future on the WACP if permafrost continues to warm^47^ and precipitation continues to increase.^48^ However, our detailed temporal analysis of lake drainage between 2000 and 2017 shows that particular years may account for the majority of lake drainage events in a given decadal‐scale time period. Future research should focus on event‐driven landscape changes on the ACP.

While several studies have focused on the timing of lake drainage in the Arctic, few have provided useful information or direct observations on the lake drainage mechanisms that can help inform future assessment of landscape change in lake‐rich regions. Our attempt at identifying lake drainage mechanisms can be improved further by conducting concerted field observations of lake sites that have a high potential to drain in the future. Understanding the potential lifespan of a lake is important for assessing its long‐term role within the ecosystem and as a freshwater source. Similarly, it is important to understand the mechanisms driving drainage events and their spatial and temporal dynamics in order to assess a lake's potential as a natural hazard and to better understand the impact of catastrophic drainage on permafrost processes, vegetation dynamics, habitat change, nutrient cycling, and hydrology both within the lake basin system and downstream.

Future studies should focus on conducting assessments of lakes that are likely to drain in the near future so that data can be collected leading up to, during, and after the lake drainage to provide better insights into the processes driving lake drainage in the Arctic. Information derived from the lake drainage gradient and potential drainage event dataset could be used to conduct detailed species‐specific studies to determine the “winners” and “losers” associated with lake drainage on the landscape^49^. In addition, the potential lake drainage dataset could be used to identify future potential hazards associated with lake drainage and ongoing land use change activities occurring in the region. A series of lakes with a high potential for drainage could be selected to monitor physical as well as biological processes leading up to and then following drainage. This type of data collection effort would provide detailed information on what may happen at the landscape scale given future projections of reduced lake extent in the Arctic. The information in the drainage dataset is also of potential interest for subsistence users and industry as sites are selected for hunting and fishing activities or drill site pads, roads, and pipeline siting, respectively.

## 6 CONCLUSIONS

We have combined multiple sources of remote sensing data available from 1955 to 2017 for the WACP in northern Alaska to identify the number of lakes larger than 10 ha that have drained completely or partially. This analysis showed that 98 lakes drained over the 62‐year period. In spite of observed climate and landscape changes occurring in the region, the decadal‐scale lake drainage rate has decreased by 39% over time. However, our analysis of annual observations of lake drainage since the year 2000 showed that 85% of the lakes drained in just two years of this 17‐year period, indicating high interannual variability in lake drainage patterns in the study region, with relationships to annual temperature and precipitation data unclear and deserving of greater investigation. The dominant lake drainage mechanism inferred in the historical lake drainage dataset was bank overtopping and/or headward erosion, occurring primarily in high‐ and low‐centered ice wedge polygon terrain. By combining observations of historical lake drainage events with lakes and topographic information derived from a 5‐m‐resolution DSM, we identified ~1900 lakes exhibiting lake and adjacent landscape characteristics that might make them prone to drainage in the future. Eighty five per cent of the lakes that drained between ca. 2000 and 2017 were identified in this lake drainage potential dataset. This dataset may prove to be a useful resource when planning for future lake drainage associated with changing climate and land use activities on the WACP of Alaska.
